# Supplementary figures and images for: Genetics of Resistance to Common Root Rot (Spot Blotch), Fusarium Crown Rot, and Sharp Eyespot in Wheat
Source: Front Genet. 2021 Jun 23;12:699342. doi: 10.3389/fgene.2021.699342 (PMC8260946; doi:10.3389/fgene.2021.699342)

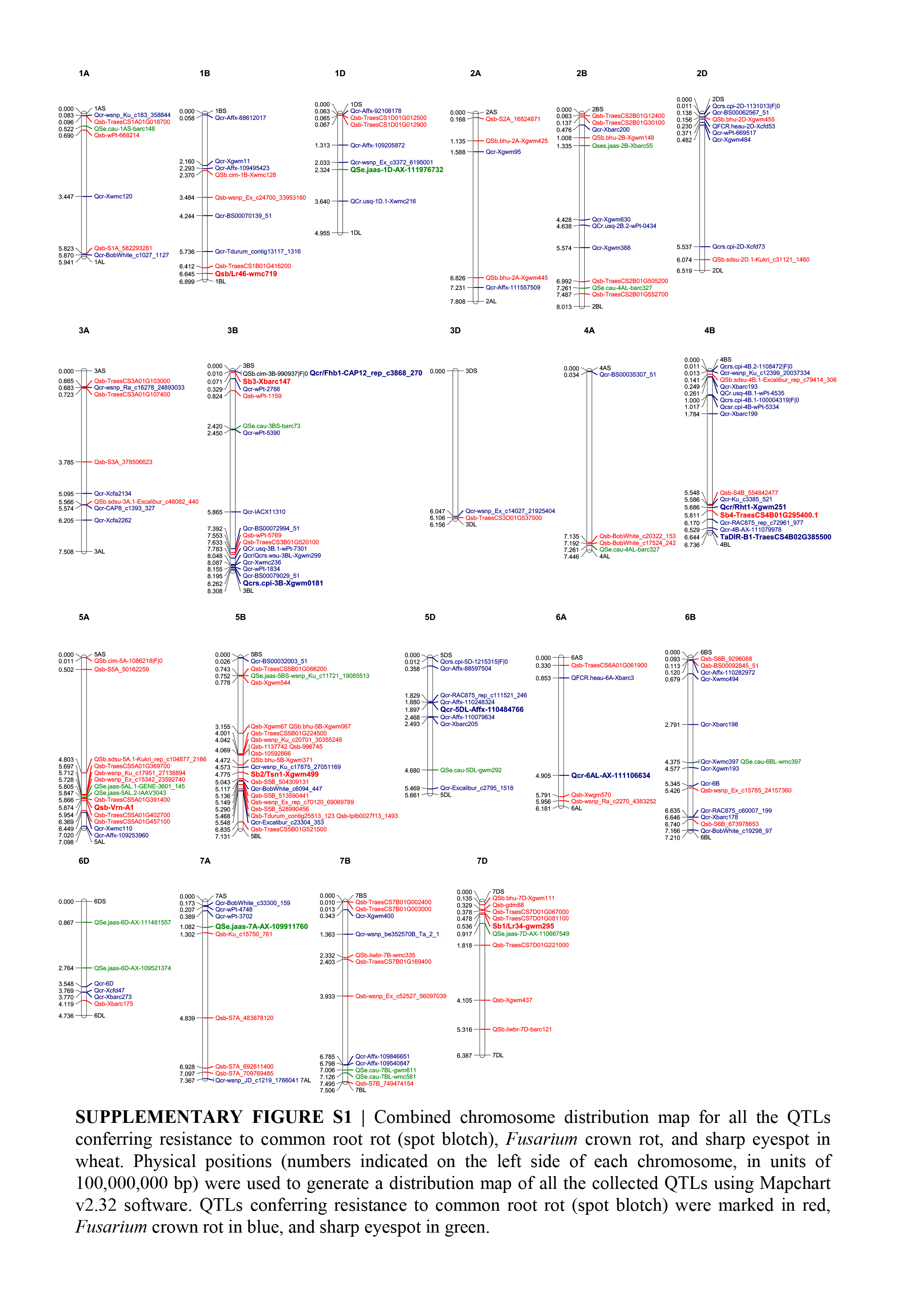

Supplement: Supplementary Figure 1 — Combined chromosome distribution map for all the QTLs conferring resistance to common root rot (spot blotch), Fusarium crown rot, and sharp eyespot in wheat. [file Image_1.JPEG]
